# Antimicrobial agents based on metal-ion zeolite materials: a multivariate approach to microbial growth inhibition

**DOI:** 10.1039/d5ra05465f

**Published:** 2025-10-01

**Authors:** Joana Guedes, Diogo B. Gonçalves, Catarina F. Rodrigues, Pier Parpot, António M. Fonseca, Cristina Almeida-Aguiar, Isabel C. Neves

**Affiliations:** a Chemistry Centre of the University of Minho (CQ-UM), Department of Chemistry, University of Minho, Campus de Gualtar 4710-057 Braga Portugal ineves@quimica.uminho.pt; b CBMA – Centre of Molecular and Environmental Biology, Department of Biology, University of Minho 4710-057 Braga Portugal cristina.aguiar@bio.uminho.pt; c CEB – Centre of Biological Engineering, University of Minho, Campus de Gualtar 4710-057 Braga Portugal

## Abstract

Bacteria are susceptible to zeolites doped with metal ions. Although the complete mode of action remains unclear, it is widely accepted that metal ions kill bacteria by inducing the production of reactive oxygen species (ROS), which are detrimental to microbial life processes. In this study, two zeolite structures, MFI and LTA, were selected as hosts for the preparation of various metal-ion zeolite materials, which were then tested for their antimicrobial activity against eight different bacterial strains—*Escherichia coli*, *Enterococcus faecalis*, *Klebsiella pneumoniae*, *Staphylococcus saprophyticus*, *Proteus mirabilis*, *Pseudomonas aeruginosa*, methicillin-sensitive *Staphylococcus aureus* (MSSA) and methicillin-resistant *Staphylococcus aureus* (MRSA)—and five yeasts—*Saccharomyces cerevisiae*, *Candida albicans*, *Candida tropicalis*, *Candida glabrata* and *Candida parapsilosis*. Minimum inhibitory concentrations (MICs) and antimicrobial efficacies (%) were determined for each material–microbe pair. In addition to comparing eukaryotic and prokaryotic models, bacterial susceptibility was assessed across differences in cell wall structure (Gram-positive *vs.* Gram-negative), growth phase (exponential *vs.* stationary), and strain type (clinical isolate *vs.* type strain). Principal component analysis (PCA) and hierarchical clustering were used to identify patterns across MIC and antimicrobial efficacy data of the antimicrobial performance of metal-ion zeolite materials. Furthermore, ANOVA-simultaneous component analysis (ASCA) was applied on a balanced *a posteriori* designed dataset to assess the contribution of experimental factors to the observed variance. To demonstrate a direct application, selected samples were preliminary tested as coatings for fruit packaging to evaluate their potential for prolonging shelf life. These findings highlight the potential of metal-ion exchanged zeolites as antimicrobial agents for healthcare and food packaging applications.

## Abstract 2

Bacteria are susceptible to zeolites doped with metal ions. PCA and ASCA were applied to identify the most effective metal-ions zeolite candidates.

## Graphical abstract

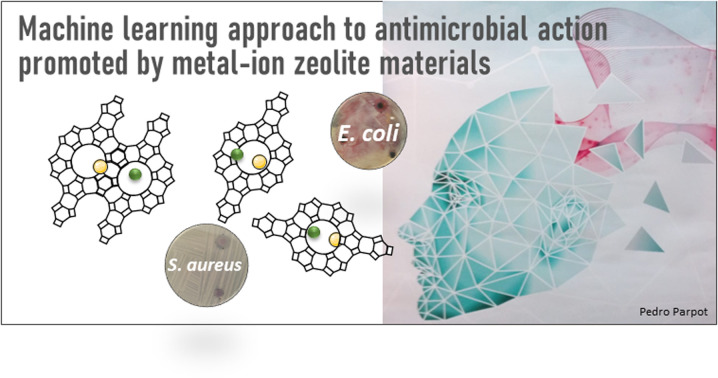

## Introduction

In recent years, the world has been confronting the alarming reality of microbial resistance to antibiotics, which is causing millions of deaths annually due to the resurgence of previously controlled infections.^1^ Antimicrobial resistance (AMR) is likely to become one of the biggest challenges of this century. Rizzello and Pompa^2^ described thoroughly the problem of antibiotic-resistant bacteria, which has been exacerbated by the significant decrease in the number of antibacterial agents that have been approved in recent decades and by increased healthcare costs.

In 2017, the World Health Organization (WHO) released a list of pathogens of global priority to guide research and development of new antibiotics, specifically targeting multidrug and extensively drug-resistant Gram-negative bacteria.^3^ This group is divided into three categories: critical microbes, including *Pseudomonas aeruginosa*, Enterobacteriaceae, *Klebsiella pneumonia*, *Escherichia coli*, *Enterobacter* spp.*, Serratia* spp., *Proteus* spp., *Providencia* spp., and *Morganella* spp.; high-priority microbes, comprising *Enterococcus faecium*, *Staphylococcus aureus* and *Helicobacter pylori*; and *Streptococcus pneumonia*, which is designated as a bacterium of medium priority. These microorganisms are commonly found in the environment.

In this work, we used some of these bacteria and well-known important yeast strains to test the antimicrobial activity of different zeolite structures based on MFI (ZSM-5, Zeolite Socony Mobil-5) and LTA (NaA, Linde Type A) modified with metal ions.

MFI and LTA zeolite structures are currently used in industry and have the potential to be used for cleaning hospital surfaces or as additives for diapers in order to prevent infections or wounds in adults. LTA (Linde Type A) zeolite is characterized by sodalite cages that are organized in a primitive cubic arrangement joined through a common 8-ring channel system, and it is used as a desiccant and as an ion-exchanger in laundry detergents.^4^ ZSM-5 is a member of the pentasil family of zeolites, distinguished by its high silica-to-alumina (Si/Al) ratio and featuring a structure consisting of oxygen-bridged, ridged sheets with 10-ring channels connecting the pentasil units.^4^ This zeolite is widely used in the petroleum industry as a heterogeneous catalyst for hydrocarbon isomerization reactions. Belonging to a broader class of materials with unique characteristics such as high cation exchange capacity, large specific surface area, shape selectivity, high adsorption capacity, and exceptional thermal and biological stability, ZSM-5 is an ideal host for various compounds in healthcare and medical applications.^5–7^ MFI and LTA zeolites can be ion-exchanged with silver, zinc or copper, which are known for their strong and broad-spectrum antimicrobial effects; especially silver,^8–10^ followed by zinc^11,12^ and copper.^13,14^ Yet, the two 3D frameworks have different ion-exchange capacity for metals, with LTA exhibiting a higher capacity due to its lower Si/Al ratio.

Antimicrobial properties of zeolites have long been tested against *Escherichia coli*^8^ as well as other bacteria such as *S. aureus* (the methicillin-sensitive and the methicillin-resistant strains, respectively, MSSA and MRSA) and *P. aeruginosa*. Yeasts, such as *Candida albicans* and *Candida tropicalis*, have been increasingly included in such antimicrobial assays.^8,9,15^ The selection of susceptible indicator strains is critical for ensuring the appropriate evaluation and application of the putative antimicrobial agent under investigation.^16,17^ Ideally, the target microbe(s) should be screened, but this is not always possible. Nevertheless, as there are several microbial features that are relevant to antimicrobial susceptibility, such characteristics should be addressed when selecting indicator strains. Cell wall structure is a well-known determinant of bacterial susceptibility to several antimicrobial agents,^17,18^ making it essential to test both Gram-positive and Gram-negative bacteria for antimicrobial susceptibility. Thus, representatives of both cell wall types were included in this study. Susceptibility differences have also been reported between laboratory reference strains and clinical isolates,^18^ highlighting the importance of incorporating both in antimicrobial assays, as done here. The microbial growth phase further influences antimicrobial activity,^19^ and this parameter was likewise examined. Finally, to account for differences in cell type, both bacteria and yeast were tested as representatives of prokaryotic and eukaryotic unicellular organisms, respectively.

Investigating these microbial characteristics across a diverse panel of test organisms can clarify whether a single indicator strain or microbial trait is sufficient to evaluate and distinguish antimicrobial efficacy. If so, this strategy may help identify the most representative strain or trait for defining a standardized susceptible reference strain. Given the number of prepared materials and studied microorganisms, the resulting data benefit greatly from the application of multivariate statistical methods to elucidate the relationship between zeolite-based materials and microbial susceptibility indicators. One of these methods, Principal Component Analysis (PCA), has been widely applied in similar contexts.^20–27^ PCA involves algebraic transformations that rotate the dataset to remove linear correlations among variables. The newly deduced features are called principal components (PCs) and capture the variance related to the original features. In this context, PCA allows zeolite samples to be grouped according to similarities in minimum inhibitory concentration (MIC) values and/or growth inhibition percentages under the tested conditions.^25–27^ More recently, ANOVA–Simultaneous Component Analysis (ASCA) has emerged as a powerful tool to assess the influence of experimental factors in structured datasets. ASCA decomposes data variance into main effects and interactions, facilitating a deeper understanding of how factors such as material concentration, growth phase, strain type and cell wall structure influence zeolite-induced microbial growth inhibition. By combining the interpretability of ANOVA with the dimensionality reduction of PCA, ASCA offers statistically robust insights into factor significance and interaction effects, particularly in datasets with replicates and design constraints.^28,29^

This study aims to identify zeolite samples with the best performance in inhibiting the growth of the tested microorganisms by applying multivariate data analysis methods, particularly PCA and ASCA. A further objective is to assess whether a limited and standardized set of susceptible indicator strains can serve as reliable proxies for evaluating the antimicrobial properties of the tested materials. Additionally, selected metal-ion zeolite materials were applied as coatings in fruit packaging, and their antibacterial activity was tested against *Escherichia coli* and *Staphylococcus aureus* at both room temperature and 4 °C.

## Experimental

### Preparation of the metal-ion zeolite materials

Seventeen metal-ion zeolite materials were prepared: ten with LTA structure and seven with MFI structure, using the typical ion-exchange method under the experimental conditions described in our previous works.^11,12,15^ The samples were prepared using different commercial zeolites in powder form: NaA from the LTA structure (Si/Al = 1.24, BCR-705, Sigma-Aldrich) and (NH_4_)ZSM-5 from the MFI structure (Si/Al = 15.0, CBV 3024E, Zeolyst International). Ion exchange was performed using aqueous solutions of MNO_3_, where M is Ag(i) (silver nitrate, AgNO_3_; Fisher Scientific), Zn(ii) (zinc nitrate, Zn(NO_3_)_2_·6H_2_O; Sigma-Aldrich) or Cu(ii) (copper nitrate, Cu(NO_3_)_2_·3H_2_O; Ridel-Haen). Sample preparation was carried out sequentially: the zeolite was first treated with an aqueous solution of the initial metal ion (M1) to promote ion exchange, followed by the introduction of the second metal ion (M2) under identical conditions, as outlined in eqn (1) and (2):^11,12,15^

1 (NH_4_)MFI + M1^*n*+^(aq) → M1(NH_4_)MFI + *n*NH_4_^+^(aq)

2 M1(NH_4_)MFI + M2^*n*+^(aq) → M1M2MFI + *n*NH_4_^+^(aq)

Briefly, the pristine zeolites were mixed with silver, zinc or copper nitrate solutions (0.01 or 0.05 M) using a metal solution/zeolite ratio of 25 mL g^−1^. After each ion-exchange treatment, all suspensions were filtered off, washed with deionized water, dried in an oven at 60 °C for 8 h, and calcined at 500 °C for 4 h under a dry air stream.

Table S1 in the SI shows all the synthesized metal-ion zeolite materials, with the respective metal (M) amount determined by ICP-AES, as well as the nomenclature used to identify the samples: M*n*1M*n*2-ZEO (*n*1 and *n*2 are the number of mmol of the first and of the second metal ion-exchanged, respectively, and ZEO refers to the pristine zeolite structure).

### Characterization methods

The metal loading of mono- and bimetal-ion zeolite materials was determined by Inductively Coupled Plasma Atomic Emission Spectrometry (ICP-AES) using an ICP-AES Horiba Jobin-Yvon model Ultima Spectrometer with the SMEWW 3120 method, after acid digestion of the samples in the “Laboratory of Analyses” of the Instituto Superior Técnico (Portugal). Some samples were characterized by X-ray photoelectron spectroscopy (XPS), which was performed at the Centro de Apoio Científico-Tecnolóxico á Investigación (CACTI) da Universidade de Vigo, Spain. Analysis of the samples was performed using a ThermoScientific K-Alpha ESCA instrument, equipped with aluminium Kα monochromatized radiation with a 1486.6 eV X-ray source. Due to the nonconducting nature of the samples, an electron flood gun was used to minimize surface charging. Neutralization of the surface charge was performed by using both a low-energy flood gun (electrons in the range 0 to 14 eV) and a low-energy argon ion gun. Photoelectrons were collected from a take-off angle of 90° relative to the sample surface. The measurement was done in Constant Analyser Energy mode (CAE) with a 100 eV pass energy for survey spectra and a 20 eV pass energy for high-resolution spectra. Surface elemental composition was determined using the standard Scofield photoemission cross sections.

### Evaluation of antimicrobial activity

#### Microbial indicator strains

The antimicrobial activity of the metal-ion zeolite materials was initially evaluated by determining their minimum inhibitory concentration (MIC) against a panel of several bacteria and yeast strains (Table S2) using the agar dilution technique.

The laboratory bacterial strains and yeast used as susceptible indicator strains were obtained from the culture collection of the Department of Biology of the University of Minho (DBUM). Bacterial clinical isolates were obtained upon consent from patients from the Hospital de São João in Oporto (HSJP, Portugal) and kindly provided by Dr^a^. Cidália Pina-Vaz.

#### Determination of minimum inhibitory concentration (MIC) of metal zeolite-based materials

The MIC of all samples (Table S1) was determined against all bacteria and yeast strains (Table S2). Bacteria were cultured in LB medium (Difco) at 37 °C and 200 rpm until the optical density at 600 nm (OD_600_) reached 0.4–0.6. Each mid-exponentially growing cell culture was serially diluted from 10^−1^ to 10^−4^ and three 5 μL-drops of each dilution were transferred to LBA (LB medium supplemented with agar 2% (w/v)) plates supplemented with different concentrations (0.1, 0.2, 0.5, 1.0 and 2.0 mg mL^−1^) of each zeolite sample (Table S1), or to single LBA plates used as control. Plates were observed after 24 h incubation at 37 °C for the presence/absence of growth, and MIC values were expressed as the lowest concentrations for which no microbial growth was detected. Assays were repeated three times, and the results were expressed as mean value ± SD.

A similar procedure was performed for yeast strains, which were cultured in YPD medium (Difco) at 30 °C and 200 rpm. Upon reaching OD_600_ 0.4–0.6, cell cultures were serially diluted from 10^−1^ to 10^−4^ and three 5 μL-drops of each dilution were transferred to YPDA (YPD medium supplemented with agar 2% (w/v)) plates supplemented with the above concentrations of each zeolite-based material (Table S1), or to YPDA alone for control. Plates were observed after 48 h incubation at 30 °C and MIC values were expressed as the lowest concentrations for which no growth was detected. Results were expressed as mean MIC value ± SD of a minimum of three assays.

#### Evaluation of antimicrobial efficacy of metal-ion zeolite materials

Yeast and bacteria were cultured in appropriate media and conditions, as already described, to the mid-exponential phase of growth (OD_600_ = 0.4–0.6). A volume of 10 mL of each microbial culture was then centrifuged at 5000 rpm for 2 min at 4 °C (ref. 11 and 12) and the pellet was washed twice with half of the volume of distilled water. In the final washing step, the pellet was resuspended in 10 mL of YPD or LB medium, depending on the microbial type, respectively, yeast or bacteria. Aliquots were then used and serially diluted from 10^−1^ to 10^−5^.

The number of yeast colony forming units (CFU) was calculated upon application of 10 μL drops of each dilution on the appropriate medium: YPDA supplemented with 0.5 and 1 mg mL^−1^ of each sample, YPDA supplemented with the basic form of pristine zeolites (NaA and ZSM5) at the same concentrations, and YPDA without supplementation for growth control. After 48 h of incubation at 30 °C, CFU were counted. Three antimicrobial assays, each with five replicates, were performed, and the results were expressed as the mean value of CFU ± SD.

The percentage of microbial growth inhibition at 0.5 and at 1 mg mL^−1^ zeolite concentrations was calculated, and the results were expressed as antimicrobial efficacy (%) according to the following equation:^12^3
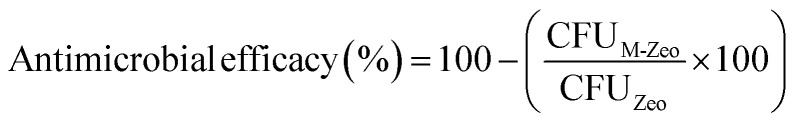
where CFU_M-ZEO_ represents the medium number of CFU mL^−1^ obtained in a given zeolite exchanged with metals, while CFU_ZEO_ is the medium number of CFU mL^−1^ observed in the pristine zeolite.

The same procedure was applied for bacteria, but LB and LBA media were used instead of YPD and YPDA, respectively, one more dilution was added (10^−6^), and the incubation conditions were at 37 °C for 24 h.

Antimicrobial assays were also performed with strains at the stationary phase (Table 1) in a procedure very similar to the one described above, with the exception of using microbial cultures directly after overnight growth. The antimicrobial efficacy of the samples against laboratory strains and clinical isolates of some bacterial species was also compared.

**Table 1:** Concentrations (mg mL^−1^) of metal-ion zeolite materials and indicator bacterial and yeast species used in antimicrobial assays to evaluate the influence of the strain type (laboratory strain (t)/clinical isolate (ci)) and growth phase (exponential (E)/stationary (S) phases)

Bacteria	Origin	Growth phase	Concentration of the material (mg mL^−1^)	Label
*E. coli*, Ec	Clinical isolate, Ec_ci_	E	0.5	**P16**
			1.0	**P17**
		S	0.5	**P18**
			1.0	**P19**
	Type strain, Ec_t_	E	0.5	**P20**
			1.0	**P21**
*MSSA*	Clinical isolate, MSSA_ci_	E	0.5	**P22**
			1.0	**P23**
		S	0.5	**P24**
			1.0	**P25**
	Type strain, MSSA_t_	E	0.5	**P26**
			1.0	**P27**
*MRSA*	Clinical isolate, MRSA_ci_	E	0.5	**P28**
			1.0	**P29**
		S	0.5	**P30**
			1.0	**P31**
*Kpl*	Clinical isolate, Kpl_ci_	E	0.5	**P42**
			1.0	**P43**
*Sts*	Clinical isolate, Sts_ci_	E	0.5	**P44**
			1.0	**P45**
*Enc*	Clinical isolate, Enc_ci_	E	0.5	**P46**
			1.0	**P47**
*Pm*	Clinical isolate, Pm_ci_	E	0.5	**P48**
			1.0	**P49**
*Pa*	Clinical isolate, Pa_ci_	E	0.5	**P50**
			1.0	**P51**
*Enf*	Clinical isolate, Enf_ci_	E	0.5	**P52**
			1.0	**P53**

**Yeast species**				
*Sc*	Type strain, Sc_t_	E	0.5	**P32**
			1.0	**P33**
*Ca*	Clinical isolate, Ca_ci_	E	0.5	**P34**
			1.0	**P35**
*Cg*	Clinical isolate, Cg_ci_	E	0.5	**P38**
			1.0	**P39**
*Cp*	Clinical isolate, Cp_ci_	E	0.5	**P40**
			1.0	**P41**

See Table S2 in the SI for further details on the strains studied in this work.

#### Evaluation of antimicrobial efficacy of paper trays coated with selected metal-ion zeolite materials

Selected zeolite samples, identified through multivariate analysis, were used to assess the antimicrobial properties of paper fruit trays coated with these materials. Commercial moulded pulp fruit packaging trays, typically used in supermarkets for transport or storage, were cut into 6 mm discs from the alveolar structure and impregnated with 5 mL suspensions of the selected samples for 24 h, followed by drying at room temperature. The antimicrobial activity of the zeolite-coated discs from the paper fruit trays was evaluated using the agar diffusion technique against the Gram-negative bacterium *Escherichia coli* CECT423 and the Gram-positive methicillin-sensitive *Staphylococcus aureus* (MSSA) (Table S2). Overnight bacterial LB cultures were used to inoculate LBA plates, which were evenly spread with bacterial suspension using sterile swabs. The prepared sample-coated discs were then placed on top of the inoculated plates with the microbial overlay. Controls included untreated discs, water-impregnated discs, and commercial antibiotic discs containing tetracycline or chloramphenicol (Becton Dickinson and Company). After 48 h incubation at room temperature or 4 days at 4 °C, the plates were examined for growth inhibition zones.

### Multivariate analysis methodology

#### Principal component analysis (PCA), ANOVA–simultaneous component analysis (ASCA), clustermaps, and correlation plots

Prior to analysis, data from the microbial growth inhibition tests were mean-centered. PCA was used for exploratory data analysis to reduce dimensionality and visualize similarities or differences between zeolite samples based on antimicrobial response. ASCA was employed to assess the significance and magnitude of experimental factors, such as zeolite structure, metal content, and microbial type, to the observed variance in antimicrobial efficacy. Since ASCA requires a balanced designed dataset, a few instances were removed *a posteriori*. The relative contribution of each factor, and the residual to the total variance, was quantified by computing the sum of squares (SSQ) for each effect matrix and expressing it as a percentage of the total SSQ of the mean-centered data matrix. Given that the considered experimental effects contain only two levels, all ASCA plots show the first dimension, which captures 100% of the explained variance. Cluster maps were generated using Euclidean distance to identify sample groupings with similar behaviour. Correlation plots were used to evaluate relationships among measured parameters across all samples. All analyses were performed using custom Python routines used in the research group.

## Results and discussion

Given the broad panel of susceptible microbial indicator strains and parameters examined, together with the diverse set of metal-ion zeolite materials investigated (17 samples in addition to two pristine zeolites), a substantial volume of experimental data was generated. The conventional approaches to data analysis, when applied to such extensive datasets, inevitably give rise to several critical questions.

Is it important to test a large panel of microorganisms in antimicrobial assays? Is there any difference between microbial susceptibility when in the stationary or exponential growing phases? Should prokaryotes and eukaryotes both be included in antimicrobial screenings? Should bacteria with different cell wall structures always be tested? Moreover, will it be possible to use a single model microbe to assess the antimicrobial properties of metal-ion zeolite materials? Lastly, how can the best antimicrobial material(s) be selected?

Data analysis is the key to understanding the behaviour of antimicrobial materials. Briefly, the strategy developed applies a multi-data decision analysis approach, which allows the evaluation of different alternatives according to data obtained from MIC and antimicrobial efficacy. This methodology can incorporate both results of MIC and percentage of inhibition and generate evidence-based data in a transparent, explicit, and deliberative mode. The main strength of this approach is the relatively high weight given to the evidence retrieved and summarized for each criterion. The method has been performed through the following steps:

(1) Selection based on the MIC response for all the metal-ion zeolite materials tested against bacteria and yeast.

(2) For each zeolite structure, selection of the samples as a function of the percentage inhibition for the tested bacteria and yeast.

(3) Data extraction from ANOVA and multivariate analysis.

(4) Selection of the best discriminatory samples.

(5) Finalization of the ranking of microbial indicator strains and of the best metal ions-zeolite material.

**Fig. 1:**
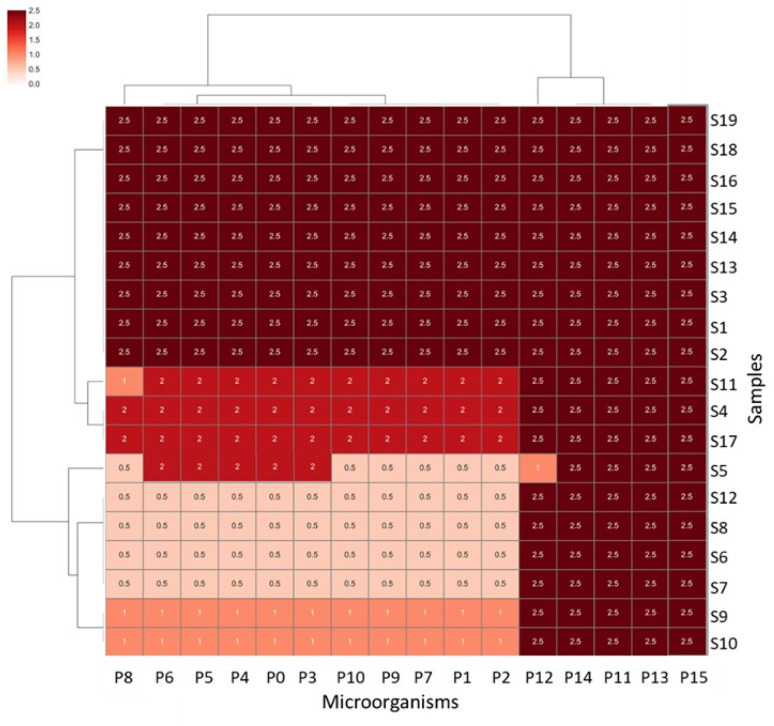
Cluster map of the MIC values (mg mL^−1^) obtained for all zeolite samples against the tested microorganisms (2.5 = MIC >2.0 mg mL^−1^). See Tables S1–S3 for detailed explanations of each zeolite sample and microbial strains, respectively.

The antimicrobial activity of metal-ion zeolite materials (see Table S1) was initially evaluated by MIC determination against a panel of several bacteria and yeast strains (Table S2) using the agar dilution technique. Fig. 1 shows the cluster map of the MIC values (Table S3) obtained for all zeolite samples against the tested microorganisms. Antimicrobial assays were conducted using metal-ion zeolite samples at concentrations up to 2.0 mg mL^−1^. If microbial growth was observed at a maximum concentration, MIC was recorded as >2.0 mg mL; however, for data analysis purposes, these cases were treated as MIC = 2.5 mg mL^−1^.

As expected, no MIC results (Table S3) were obtained for samples S1 (ZSM5) and S2 (NaA) (Table S1), showing that these pristine zeolites do not present antimicrobial activity against the studied strains in the tested concentration range (MIC = 2.5 mg mL^−1^).^9,11,12^

From the results, different observations were made: first, the presence of metals significantly enhances the antimicrobial properties of the samples. Secondly, the most effective results were achieved when the LTA structure was used as the pristine zeolite. Finally, both zeolite structures exhibit nearly identical behaviour against yeast (**P11–P15**; Table S2), with MIC >2.0 mg mL^−1^, except Ag_2.5_A (S5, 5.2 wt%_Ag_), which demonstrated activity against the clinical isolate *C. parapsilosis* (**P12**, Cp_ci_) at 1 mg mL^−1^ (Table S3). Apparently, for these zeolite materials, the use of yeasts as susceptibility indicators may not be necessary, although including both bacterial and fungal models in antimicrobial screenings remains valuable for assessing the spectrum, efficacy, and safety of antimicrobial agents across diverse pathogens and cell types.

**Fig. 2:**
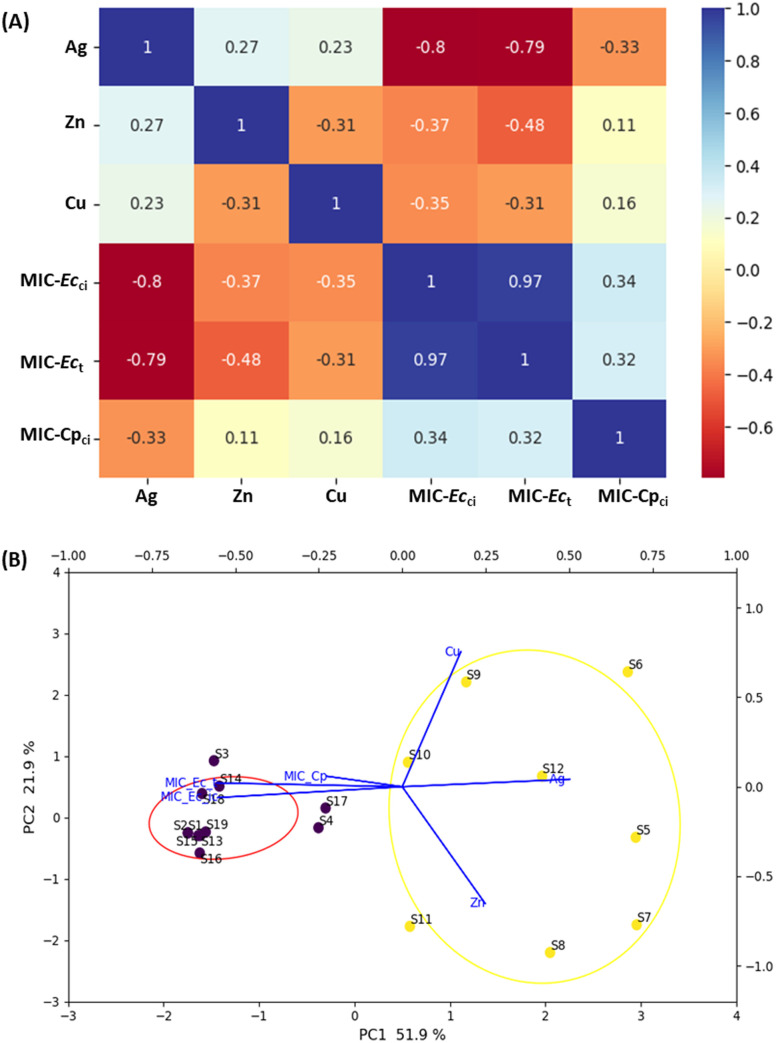
(A) Correlation plot of machine learning variables using hierarchical clustering methods for the range of MIC values against strains **P8** and **P7** (MIC-Ec_t_ and MIC-Ec_ci_) and **P12** (MIC-Cp_ci_). (B) Bi-plot graphic PC1 *vs.* PC2 of the MIC values (mg mL^−1^) obtained for all zeolite samples against the selected bacteria (**P7** and **P8** or Ec_ci_ and Ec_t_, respectively) and yeast (**P12** or -Cp_ci_).

To identify the most effective samples against the tested microorganisms based on MIC values, two strains from the same bacterial species (*E. coli*, Ec_t_ (**P8**), and Ec_ci_ (**P7**)) and one yeast (**P12**, Cp_ci_) (Table S2) were chosen as representatives to reflect the range of MIC values (Table S3). Fig. 2A illustrates the correlation obtained through machine learning for the selected strains.

As expected, a high correlation (0.97) was observed between Ec_t_ (**P8**) and Ec_ci_ (**P7**), as they represent the same bacterial species but originate from different sources. **P8** is a type strain (DBUM), while **P7** is a clinical isolate obtained from the urine of a patient at Hospital de São João in Oporto, Portugal. Yeast strain **P12** is a clinical isolate obtained from a vaginal specimen and, unlike *E. coli*, is a eukaryotic rather than a prokaryotic microbe.

Coefficient correlation values close to one indicate a strong correlation between the variables, while correlation values close to −1 show that the variables are inversely proportional. Higher correlation values |0.80 to 0.33| were found between the silver-containing samples and the three microorganisms, which prove the importance of silver in decreasing the MIC values.

PCA was utilised to obtain a lower-dimensional representation of the data from the same three microorganisms representing the range of MIC values (Fig. 2B and Table S3).

PC1 and PC2 explain together 73.8% of the system's variance of the MIC values, and 2 clusters were identified. Eight samples based on the LTA structure (S5 to S12, see Tables S1 and S3) exhibited lower MIC values against the selected microorganisms (Table S2). The other cluster, with the remaining samples, presents the highest MIC values. Ag_2.5_A (S5, 5.20 wt%_Ag_) is the best sample against the yeast, with all the other samples presenting the same MIC value (>2.0 mg mL^−1^). In the case of the bacterium, either the type strain or the clinical isolate, the lowest MIC values were obtained for S5 to S8 and S12, (MIC = 0.5 mg mL^−1^) and against both strains, followed by S9 ≈ S10 (MIC = 1 mg mL^−1^) and S11, with different MIC values for **P7** (2 mg mL^−1^) and for **P8** (1 mg mL^−1^).

This behaviour is similar for the other bacteria studied, with the LTA samples showing the best results: Ag_2.5_A (S5), Ag_2.5_Cu_0.5_A (S6, 5.10 wt%_Ag_/4.50 wt%_Cu_), Ag_2.5_Zn_0.5_A (S7, 5.07 wt%_Ag_/3.30 wt%_Zn_), Ag_0.5_Zn_2.5_A (S8, 2.10 wt%_Ag_/4.00 wt%_Cu_), Ag_0.5_Cu_2.5_A (S9, 2.10 wt%_Ag_/4.40 wt%_Cu_), Ag_0.5_Cu_0.5_A (S10, 0.70 wt%_Ag_/2.00 wt%_Cu_), Ag_0.5_Zn_0.5_A (S11, 2.10 wt%_Ag_/3.00 wt%_Zn_) and Cu_0.5_Ag_0.5_A (S12, 1.40 wt%_Cu_/4.50 wt%_Ag_). However, the bimetal-ion LTA samples Ag_2.5_Cu_0.5_A (S6), Ag_2.5_Zn_0.5_A (S7), Ag_0.5_Zn_2.5_A (S8) and Cu_0.5_Ag_0.5_A (S12) are the most active according to the lowest values of MIC (0.5 mg mL^−1^) against the bacteria *E. faecalis* clinical isolate (**P1**, Enf_ci_), *K. pneumoniae* (**P2**, Kpl_ci_), *S. saprophyticus* (**P3**, Sts_ci_), *Pr. mirabilis* (**P4**, Pm_ci_), *P. aeruginosa* (**P5**, Pa_ci_), *E. cloacae* (**P6**, Enc_ci_), *S. aureus* type strain and clinical isolate (**P10**, MSSA_t_ and **P9**, MSSA_ci_), and the methicillin-resistant *S. aureus* (**P0**, MRSA_ci_) (Table S3). These results confirm that the bimetal-ion LTA samples with lower amounts of silver are active against the bacteria, as is the case for S8 and S9, with 2.1 wt% silver.

In the case of the MFI structure, samples S13 to S19 show little variance in terms of antibacterial activity (Fig. 1 and Table S3). In fact, only Cu_0.5_Ag_0.5_ZSM-5 (S17, 0.60 wt%_Cu_/0.33 wt%_Ag_), a bimetal-ion MFI material with silver as the second introduced metal, shows a lower MIC value (2 mg mL^−1^) than the remaining samples, which display MIC >2.0 mg mL^−1^ against all the bacteria. Similar to almost all the LTA-zeolite materials, MFI-based samples show the same behaviour and the highest MIC values (>2.0 mg mL^−1^) against the tested yeast strains. Notably, in the bimetal-ion zeolite materials, the silver content is significantly reduced compared to that in the monometal zeolite, highlighting the effective synergy of Ag with Zn or Cu and the potential for cost reduction without compromising performance. In our work, we demonstrated that the antimicrobial activity of samples containing both silver (Ag) and zinc (Zn) arises from a synergistic interaction between the two metals, specifically related to their valence states and spatial distribution within the zeolite framework.^12^

**Fig. 3:**
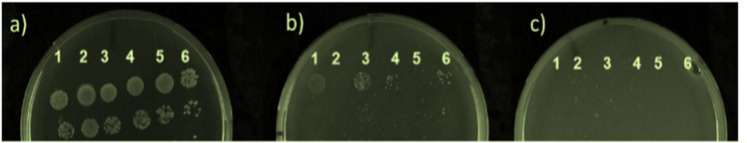
Details of an antimicrobial activity assay against (1) MRSA (**P3**), (2) *P. aeruginosa* (**P1**), (3) *En. cloacae* (**P5**), (4) *S. saprophyticus* (**P4**), (5) *Pr. mirabilis* (**P2**) and (6) *E. coli* (**P6**) in an LBA medium (a) and in LBA supplemented with (b) 0.5 mg mL^−1^ and (c) 2.0 mg mL^−1^ of Ag_2.5_A (S5).

Antimicrobial assays based on MIC determination do not always allow a clear differentiation of the zeolite materials, as microorganisms can display different responses to materials with identical MIC values (Fig. 3). For this reason, the antimicrobial efficacy of the zeolite-based materials was also evaluated in this work. From Fig. 3, we observe that the number of colonies decreases very differently with increasing Ag_2.5_A concentration. Viable cells of *P. aeruginosa* (**P1**) and *Pr. mirabilis* (**P2**) were absent in the presence of 0.5 mg mL^−1^ of this sample (S5) (Fig. 3b), being with this concentration being the MIC of S5 against **P1** and **P2**. The MIC of S5 is 2.0 mg mL^−1^ against the remaining tested bacteria, as no viable bacterial cells were observed at this concentration (Fig. 3c). Yet, at a concentration of 0.5 mg mL^−1^ of S5, differential bacterial responses were observed, with **P4** showing greater inhibition than **P6**, as indicated by a lower number of colony-forming units (CFUs) on the plate (Fig. 3b).

Therefore, following MIC determination, the antimicrobial efficacy of each material was evaluated against the same panel of microbes. The antimicrobial properties were assessed in different microbial growth phases by testing some microbes in both exponential and stationary phases of growth, and with different strain types; namely, laboratory strains and clinical isolates of the same species (Table 1). Different bacterial cell wall structures were also considered, including Gram-positive and Gram-negative bacteria in the panel of indicator susceptible strains. Table 1 summarizes the microorganisms used and the conditions tested for bacteria and yeast.

**Fig. 4:**
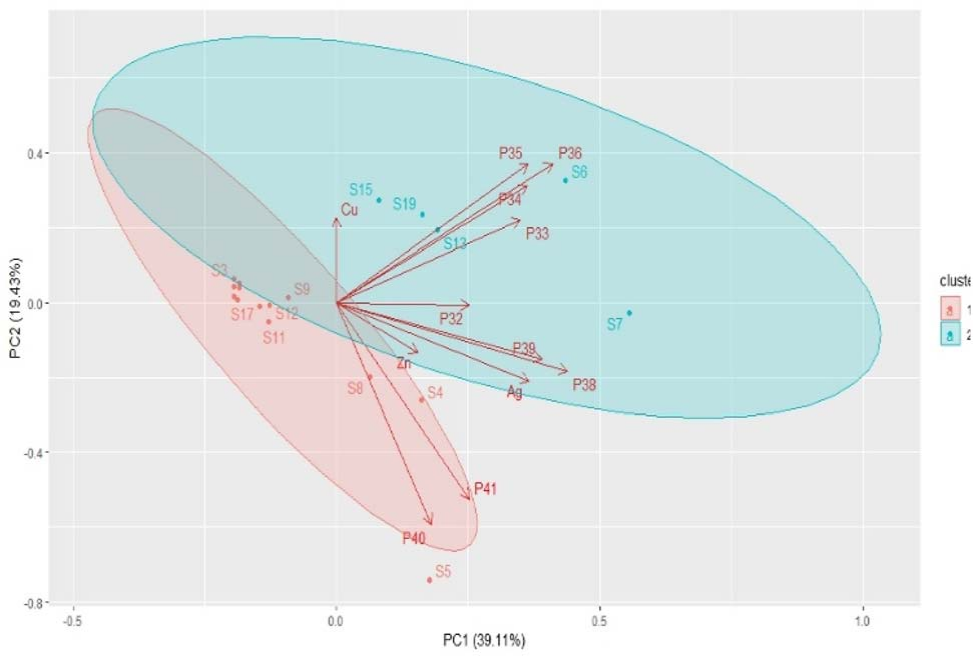
Bi-plot graphic PC1 *vs.* PC2 for the antimicrobial efficacy of all the metal ion-zeolite materials against yeast strains in the exponentially growing phase (**P32–P41**; Table S2).

The antimicrobial efficacy of all metal-ion zeolite materials was evaluated against the full panel of microorganisms using a multivariate approach. The results, expressed as PC scores in Fig. 4, show the obtained antimicrobial efficacies of the metal-ion zeolite samples prepared from both structures against yeast strains. Four yeast clinical isolates, Ca_ci_, Ct_ci_, Cg_ci_, and Cp_ci_, and a type strain, Sc_t_, were used to select the best metal-ion zeolite materials. Among the tested structures, LTA proved to be the most effective, with the Ag-containing samples exhibiting the highest performance.

Two clusters are identified (Fig. 4): in cluster 1, most of the samples are connected to the yeast Cp_ci_ at both sub-MIC concentrations of 0.5 (**P40**) and 1.0 mg mL^−1^ (**P41**). In cluster 2, five samples are linked to the other yeast studied. In this case, PC1 and PC2 explain together 58.5% of the system's variance in the exponential growth phase.

For Cp_ci_, the best antimicrobial efficacies at 0.5 (**P40**) and 1.0 mg mL^−1^ (**P41**) were obtained with Ag_2.5_A (S5), and the trend observed is S5 > S7 > S9 > S13. For Ca_ci_, the main causative agent of candidiasis, the primary fungal infection in adults and paediatric patients,^30^ antimicrobial efficacy could be scaled as S7 (Ag_2.5_Zn_0.5_A) > S15 > S19 > S13 > S6, and for the best sample, S7, 0.5 mg mL^−1^ is enough to achieve the best performance (**P34**).

For Cg_ci_, the highest tested concentration yielded the best result (**P39**) with S13 (Ag_0.5_Zn_0.5_ZSM-5, 0.50 wt%_Ag_/0.10 wt%_Zn_). The antimicrobial performance of the zeolite samples for Cg_ci_ can be ranked as S13 > S5 > S7 > S4 > S6 > S9 > S15. In contrast, for Ct_ci_, S6 (Ag_2.5_Cu_0.5_A, 5.10 wt%_Ag_/4.50 wt%_Cu_) demonstrated the best performance at 0.5 mg mL^−1^ (**P36**), with the ranking as follows: S6 > S19 > S7 > S13 > S15.

Finally, for the model eukaryotic microbe *Saccharomyces cerevisiae* (Sc_t_), the type strain, the best sample is S8 (Ag_0.5_Zn_2.5_A) at 1.0 mg mL^−1^ (**P33**), followed by S4 > S6 > S7 > S13 ≈ S15. Generally, samples prepared with the LTA structure show better activity against yeasts than those prepared with MFI zeolite. These results suggest that, for eukaryotic cells, sample selection should be guided by overall antimicrobial efficacy rather than solely by MIC values (Fig. S1).

To decompose the influence of strain type, Gram type, microbial growth phase, and material concentration on the antimicrobial efficacy of the metal-ion zeolite samples, ASCA was applied (Fig. 6A–D). This approach enabled clear visualization of the relative contribution of each experimental factor (Table 1) while identifying the zeolite sample most responsive to each condition (Tables S1 and S4).

Due to the limitations in the origin of the microorganisms, the dataset was balanced by including both Gram-negative and Gram-positive strains. The Gram-negative (G−) group included *Escherichia coli* (*E. coli* type strain, Ec_t_, and clinical isolate, Ec_ci_), while the Gram-positive (G+) strains were methicillin-sensitive *Staphylococcus aureus* (type strain MSSA_t_ and clinical isolate, MSSA_ci_). The remaining Gram-negative (G−) bacteria (*Klebsiella pneumoniae* (Kpl_ci_), *Proteus mirabilis* (Pm_ci_), and *Pseudomonas aeruginosa* (Pa_ci_)), as well as the Gram-positive (G+) strains (*Enterococcus cloacae* (Enc_ci_), *Staphylococcus saprophyticus* (Sts_ci_), methicillin-resistant *Staphylococcus aureus* (MRSA_ci_), and *Enterococcus faecalis* (Enf_ci_)), were all clinical isolates obtained from the Hospital de São João in Oporto, Portugal.

**Fig. 5:**
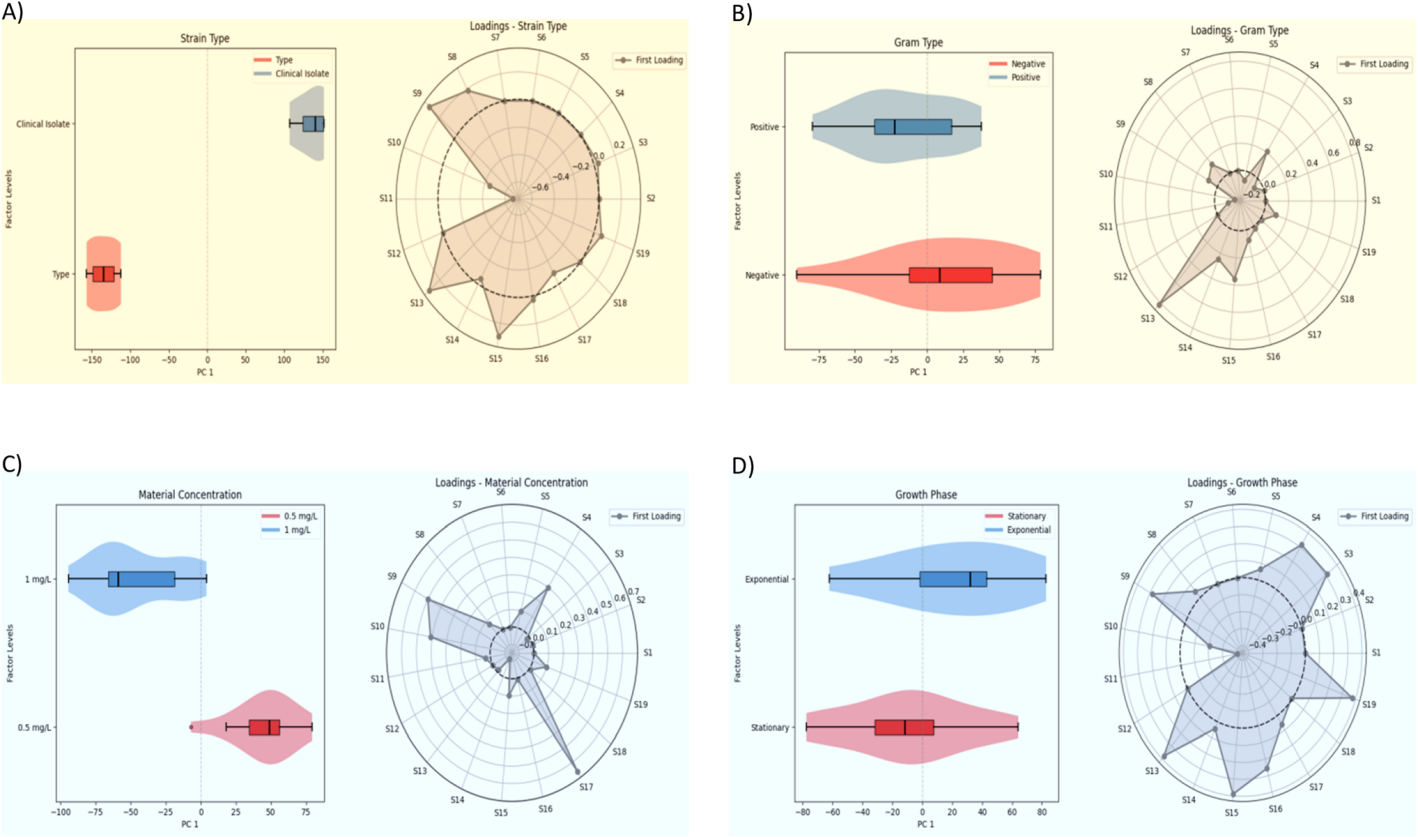
(A) Influence of strain type (clinical isolate *vs.* type strain), (B) bacterial cell wall structure (Gram-positive *vs.* Gram-negative), (C) sample concentrations (0.5 mg mL^−1^*vs.* 1.0 mg mL^−1^) and (D) microbial growth phase (exponential *vs.* stationary) on the antimicrobial efficacy of metal-ion zeolite materials evaluated by ASCA. Left and right panels show score and loadings plots, respectively. Due to the microorganism's availability, Fig. 6A dataset was balanced *a posteriori* by ensuring an equal number of instances for both levels of type strain. The corresponding dataset can be found in Table S5.

Thus, Fig. 5A shows the ASCA score plot for strain type (clinical isolate *vs.* type strain) along PC1. The distribution for clinical isolates is centred slightly on the positive side of PC1, while type strains are positioned on the negative side relative to the origin. These findings indicate that the strain type contributes modestly to the observed variability in antimicrobial efficacy, with the clinically isolated strains generally exhibiting higher susceptibility under the tested conditions. This is consistent with previous works suggesting that type strains, despite often lacking resistance elements present in clinical isolates, can exhibit lower susceptibility under certain testing conditions due to differences in growth dynamics and stress responses, resulting in clinical isolates appearing more responsive to antimicrobial agents *in vitro*.^31,32^

The loading plot for this panel highlights S13 (Ag_0.5_Zn_0.5_ZSM-5, 0.50 wt%_Ag_/0.10 wt%_Zn_), S11 (Ag_0.5_Zn_0.5_A, 2.10 wt%_Ag_/3.00 wt%_Zn_), and S9 (Ag_0.5_Cu_2.5_A, 2.10 wt%_Ag_/4.40 wt%_Cu_) as the samples contributing most to the variance under the strain type factor. These samples showed clear differences in antimicrobial activity against type strains and clinical isolates, likely reflecting the influence of Cu- and Zn-modified frameworks on strain-specific susceptibility profiles. Thus, depending on the intended application, particularly in clinical contexts, it may be essential to evaluate zeolite samples against both strain types rather than relying on only one.

Fig. 5B presents the ASCA score plot for bacterial cell wall type (Gram-positive *vs.* Gram-negative). In this analysis, Gram-positive (G+) strains are located on the negative side of PC1, whereas Gram-negative (G−) strains are positioned on the positive side relative to the origin, indicating a trend toward higher susceptibility among Gram-negative bacteria.

This observation contrasts with the conventional view that Gram-positive bacteria, lacking the outer membrane of Gram-negative bacteria, are generally more permeable to antimicrobial agents. Instead, under the tested conditions, Gram-negative strains exhibited a trend toward higher susceptibility, suggesting that factors unrelated to the outer membrane permeability may influence the observed antimicrobial efficacy. This observation is consistent with previous studies showing that Gram-negative bacteria can be, under certain antimicrobial treatments, more susceptible than Gram-positive strains,^33^ despite the traditional view that the outer membrane of Gram-negative cells provides enhanced protection.

The loading plot identifies S13 (Ag_0.5_Zn_0.5_ZSM5, 0.50 wt%_Ag_/0.10 wt%_Zn_), S15 (Ag_0.5_Zn_2.5_ZSM5, 0.50 wt%_Ag_/0.20 wt%_Zn_), and S4 (Ag_0.5_A, 3.30 wt%_Ag_) as the main contributors under the Gram type factor. The prominence of these Ag- and Zn-zeolites based on the MFI structure suggests they capture the subtle differences in cell envelope structures between G+ and G− bacteria, affecting antimicrobial response.

Fig. 5C displays the ASCA score plot for material concentration (0.5 mg mL^−1^*vs.* 1.0 mg mL^−1^), revealing a clear separation along PC1. The 0.5 mg mL^−1^ condition is positioned on the positive side of PC1, while 1.0 mg mL^−1^ is positioned on the negative side, reflecting a strong concentration-dependent effect, with lower material concentrations leading to increased antimicrobial activity across the tested samples. This counterintuitive trend may be attributed to phenomena such as concentration-dependent aggregation or self-quenching at higher doses, which can diminish the bioavailability and effectiveness of the antimicrobial agents despite their increased nominal concentration.^15,34^

The loading plot highlights S17 (Cu_0.5_Ag_0.5_ZSM-5, 0.60 wt%_Cu_/0.33 wt%_Ag_), S9 (Ag_0.5_Cu_2.5_A, 2.10 wt%_Ag_/4.40 wt%_Cu_), and S10 (Ag_0.5_Cu_0.5_A, 0.70 wt%_Ag_/2.00 wt%_Cu_) as the top contributors, indicating these Cu- and Ag-modified materials display pronounced concentration-dependent antimicrobial activity across the tested conditions.

Finally, Fig. 5D evaluates the growth phase (exponential *vs.* stationary), showing that the exponential phase is positioned on the positive side of PC1, whereas the stationary phase is on the negative side relative to the origin. This suggests that the growth phase contributes to the variance in the antimicrobial efficacy, with cells in the exponential phase tending to be more susceptible to metal-ion zeolite samples, consistent with their higher metabolic activity and previously reported differential susceptibility.^35^

The loading plot for the growth phase shows S19 (Ag_0.5_ZSM5, 0.84 wt%_Ag_), S15 (Ag_0.5_Zn_2.5_ZSM5, 0.50 wt%_Ag_/0.20 wt%_Zn_), and S13 (Ag_0.5_Zn_0.5_ZSM5, 0.50 wt%_Ag_/0.10 wt%_Zn_) as the samples contributing most to variance under this factor. This indicates that these AgZn-MFI structures exhibit distinct activity patterns depending on whether bacteria are in the exponential or stationary phase, potentially reflecting interactions with metabolic state and membrane properties.

The remaining samples exhibit good antibacterial performance, with those prepared using the LTA structure (S5, S6, S7, S8, and S12) showing the highest efficacy.

The ASCA model ascribes the relative contribution of each experimental factor to the overall variance in antimicrobial efficacy, as displayed in Table 2.

**Table 2:** Percentage of variance in antimicrobial efficacy explained by experimental factors according to ASCA decomposition

Mean	Material concentration	Strain type	Growth phase	Gram type	Residuals
86.7	2.0	2.4	2.2	0.8	10.7

The grand mean value explains the majority of the variance (86.6%), while strain type (2.4%) and growth phase (2.2%) account for the largest portions among the tested effects. Material concentration (2.0%) and Gram type (0.8%) contribute less, and residuals represent 10.7% of the variance. These results confirm the modest but detectable influence of the studied factors, consistent with the separation patterns observed in the score and loading plots. Although secondary in magnitude, such effects remain relevant for guiding antimicrobial screening: growth phase and strain background introduce measurable variability, whereas Gram type and concentration appear to play smaller roles. This indicates that microbial panels can be rationally streamlined while retaining susceptibility to the most influential biological conditions.

**Fig. 6:**
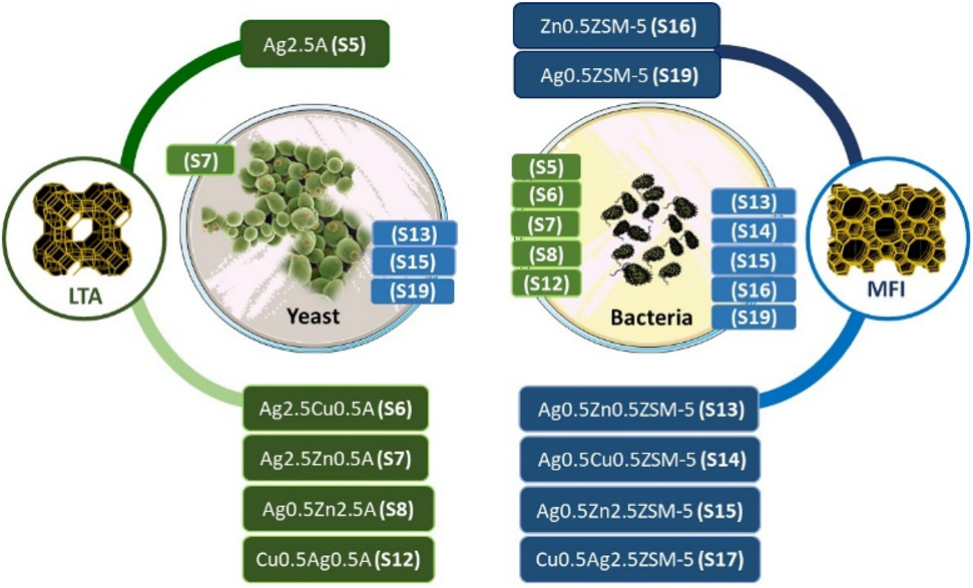
Schematic of a selection of the best zeolite samples for bacterial and yeast strains.

After this study, we can conclude and identify the best zeolite samples for the inhibition of the tested microorganisms (Fig. 6). In general, the LTA structure seems to be the best option to achieve the best antimicrobial efficacy against all the G− and G+ bacteria studied, regardless of the sample concentration used. For Gram-negative bacteria, S5 (Ag_2.5_A), S6 (Ag_2.5_Cu_0.5_A), S7 (Ag_2.5_Zn_0.5_A) and S12 (Cu_0.5_Ag_0.5_A) are the best samples, whereas for Gram-positive bacteria, S6 (Ag_2.5_Cu_0.5_A), S7 (Ag_2.5_Zn_0.5_A), S8 (Ag_0.5_Zn_2.5_A) and S12 (Cu_0.5_Ag_0.5_A) consistently exhibited superior performance and demonstrated broader effectiveness regardless of the concentration used. In the case of the MFI structure, the best samples are three with two metals, S14 (Ag_0.5_Cu_0.5_ZSM-5), S15 (Ag_0.5_Zn_2.5_ZSM-5), and S17 (Cu_0.5_Ag_0.5_ZSM-5), and two with one metal, S16 (Zn_0.5_ZSM-5) and S19 (Ag_0.5_ZSM-5).

In the case of the LTA zeolite, the best monometal-ion LTA sample is S5 (Ag_2.5_A), which shows the best performance against MRSA, *E. coli* and MSSA. The bimetal-ion samples S12 (Cu_0.5_Ag_0.5_A), S8 (Ag_0.5_Zn_2.5_A) and S7 (Ag_0.5_Zn_0.5_A) show the best results for bacteria (the same bacteria), but only the Ag_2.5_Zn_0.5_A (S7) sample was equally active against yeast (particularly Cg_ci_ and Ca_ci_).

On the other hand, for the monometal-ion MFI samples, the best results were obtained with S19 (Ag_0.5_ZSM5) against bacteria (MRSA, *E.coli* and MSSA) and yeast (Cp_ci_, Ct_ci_ and Sc_ci_) but the bimetal-ion LTA samples S13 (Ag_0.5_Zn_0.5_ZSM-5), S14 (Ag_0.5_Cu_0.5_ZSM-5) and S15 (Ag_0.5_Zn_2.5_ZSM-5) were the best against bacteria (the same bacteria) and yeast (Ca_ci_ and Cg_ci_).

However, when we take into account the amount of metal in the samples (see Table S1), the best LTA sample is S8 (Ag_0.5_Zn_2.5_A), which presents 2.10 wt% of Ag and 4.0 wt% of Zn. In the case of the MFI zeolite, both bimetal-ion zeolite samples S13 and S15 are similar and show the best performance, with both having the same amount of the two metals.

Some of the bimetal-ion samples with the best antimicrobial activity, such as S15 (Ag_0.5_Zn_2.5_ZSM-5) and S17 (Cu_0.5_Ag_0.5_ZSM-5) from the MFI structure, and S11 (Ag_0.5_Zn_0.5_A) and S12 (Cu_0.5_Ag_0.5_A) from the LTA structure, were analysed by XPS.

**Fig. 7:**
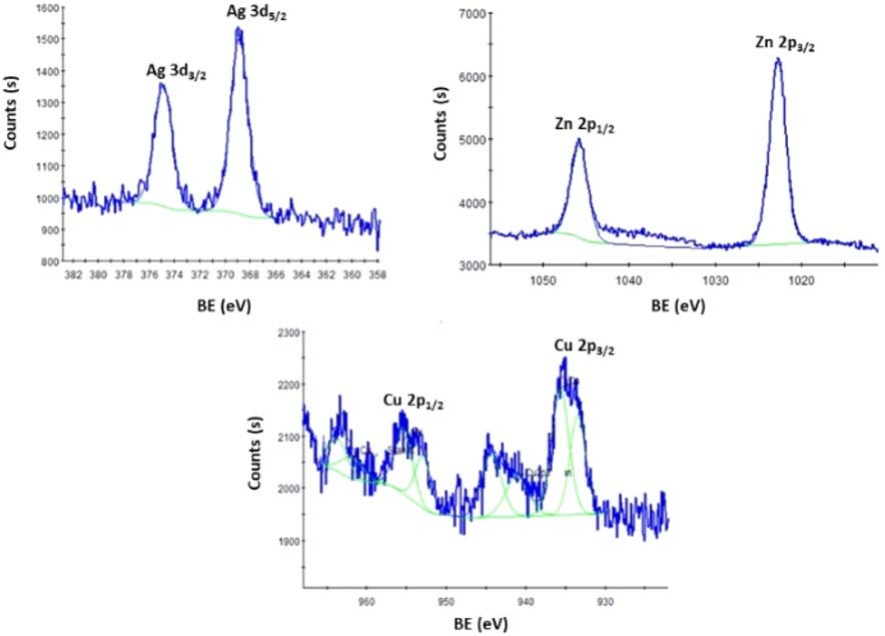
High-resolution XPS spectra of Ag 3d, Zn 2p and Cu 2p regions from samples S17, S15 and S12.

The XPS survey spectra of the samples revealed the presence of typical zeolite elements, including oxygen (O 1s, a strong peak at 532–535 eV), silicon (Si 2p at 102–104 eV), and aluminium (Al 2s at 117–118 eV). These correspond to the characteristic three-dimensional arrangement of tetrahedral units [SiO_4_] and [AlO_4_]^−^, interconnected through bridging oxygen ions. Additionally, sodium (Na 1s at 1072–1075 eV) was detected, indicating sodium's presence within the zeolite framework. The spectra also revealed zinc (Zn 2p at 1022.0–1046.0 eV), copper (Cu 2p at 933.0–936 eV) and silver (Ag 3d at 368.0–369.0 eV), in the different samples, confirming the incorporation of these metal ions into the zeolite structures. Fig. 7 shows the high-resolution XPS spectra of Cu 2p, Zn 2p and Ag 3d regions from the different samples.

The high-resolution XPS spectra of the metal-ion regions of the samples showed a closely spaced doublet with 2 main peaks assigned to Ag 3d_5/2_ and Ag 3d_3/2_, Cu 2p_3/2_ and 2p_1/2_, Zn 2p_3/2_ and Zn 2p_1/2_ regions, which correspond to emitted photoelectrons of the 3d and 2p orbitals, respectively.^12,36,37^

The two peaks of Cu 2p_3/2_ and 2p_1/2_ show binding energies (BE) close to 933.44 eV and 935.63 eV for S12 and can be ascribed to Cu(0) and Cu(ii), respectively, with a higher contribution of the Cu^2+^ (75%).^36,37^

In the case of Zn, two peaks corresponding to Zn 2p_3/2_ and Zn 2p_1/2_ are observed at BEs of approximately 1022.76 eV and 1045.74 eV, respectively, for S11. These peaks can be attributed to Zn^2+^ and Zn^0^ species, with Zn^2+^ contributing predominantly.^12,36,37^ Finally, the Ag 3d region shows two peaks with BEs at 368.88 and 374.87 eV and can be attributed to Ag(i) and Ag(0), respectively. In this case, the Ag^+^ species also has a large contribution.^12,36,37^

Table 3 summarizes the identification of metal species and the surface amount. Silver has lower amounts and zinc has higher amounts on the surface of the prepared samples, which means that a very small amount of metal is accessible to the microorganisms.

**Table 3:** XPS results: identification of binding energy (BE), oxidation state and their contribution, and the atomic percentage of the metals on the surface of zeolite samples S11, S12, S15 and S17

	High-resolution XPS spectra in the Zn 2p, Cu 2p and Ag 3d regions (BE) and relative contribution (%)			
Samples	Zn 2p (eV); (%)	Cu 2p (eV); (%)	Ag 3d (eV); (%)	M (at%)
S11	1022.81	—	368.96	0.62 (Ag)
	Zn(0); 0		Ag(i); 100	
	1045.74		374.92	2.82 (Zn)
	Zn(ii); 100		Ag(0); 0	
S12	—	933.44	369.44	0.38 (Ag)
		Cu(0); 25	Ag(i); 100	
		935.63	375.45	0.82 (Cu)
		Cu(ii); 75	Ag(0); 0	
S15	1022.76	—	369.06	0.14 (Ag)
	Zn(0): 0		Ag(i); 100	
	1045.60		375.41	<0.05 (Zn)
	Zn(ii); 100		Ag(0); 0	
S17	—	933.50	368.88	0.40 (Ag)
		Cu(0); 0	Ag(i); 100	
		935.10	374.87	<0.05 (Cu)
		Cu(ii); 100	Ag(0); 0	

For samples S11 and S12, which were prepared with LTA, the surface silver content is approximately 29.5% and 8.5%, respectively. This indicates that, in the sequential process, the amount of metal incorporated depends on which metal is introduced first. The higher surface silver content enhances the antimicrobial activity of sample S11. For samples S15 and S17, prepared with MFI, the same behaviour is observed for both samples. In S15, silver represents 28% of the surface content, whereas in S17 it is 55%.

The distribution of metal ions within the framework is governed by the sequence of ion introduction as well as the zeolite topology. The interplay of these parameters directly influences ion exchange efficiency and metal localization, and, consequently, the antimicrobial performance of the resulting materials.

Taking into account these results, samples S12 (Cu_0.5_Ag_0.5_A), S7 (Ag_2.5_Zn_0.5_A) and S19 (Ag_0.5_ZSM-5) were used to assess the antimicrobial properties for the semi-rigid alveolar structure of fruit transport and storage containers. It is common knowledge that the transport and storage of fruits is frequently compromised by microbial contamination, especially during the delivery of these perishable foods to the public. The development of smart packages against microbial contamination offers an alternative approach that is of great interest to help control post-harvest diseases. The incorporation of metal-ion zeolite materials with antimicrobial properties in the semi-rigid alveolar structure for the transport and storage of fruits could allow the preservation of fruit and maintain safety and quality by inhibiting/reducing the growth of microorganisms.^11,15,38^

**Fig. 8:**
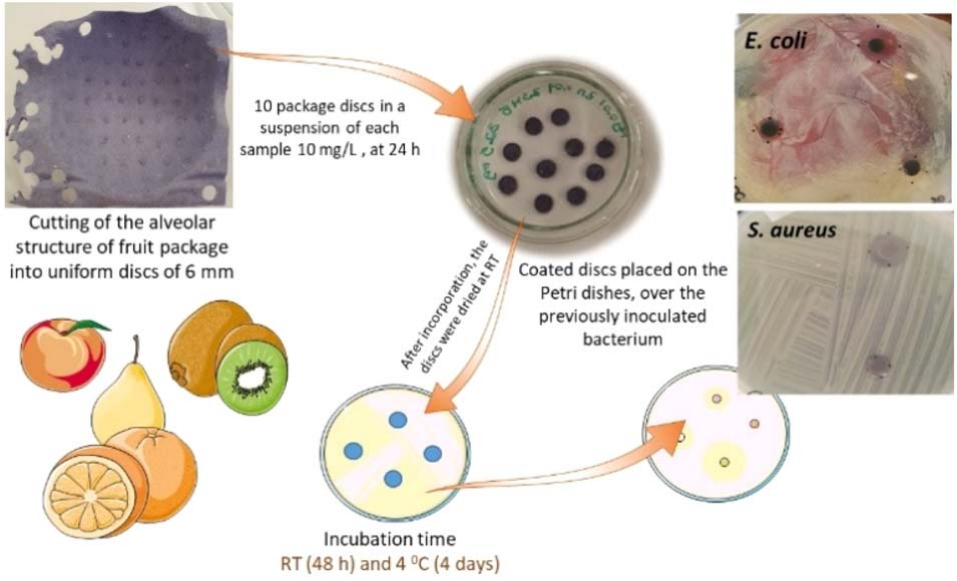
Scheme of the preparation of the assays with the metal-ion zeolite incorporated fruit package discs over an overlay of *E. coli* and *S. aureus*.

The selected samples, S7, S12, and S19, were incorporated into the fruit packaging material, and the antibacterial activity of this functionalized storage material was assessed against *Escherichia coli* and *Staphylococcus aureus* (MSSA) at room temperature and 4 °C (Fig. 8).

The results were assessed by the presence or absence of an inhibition halo, with (+) indicating the formation of growth inhibition zones, and (−) representing its absence (Table 4).

**Table 4:** Results of antimicrobial assays for coated discs against *E. coli* and *S. aureus* after incubation at room temperature

Sample	*E. coli*	*S. aureus*
NaA	−	−
ZSM-5	−	−
Ag_0.5_Zn_0.1_A (S7)	+	+
Cu_0.5_Ag_0.5_A (S12)	+	+
Ag_0.5_ZSM-5 (S19)	+	+

**Fig. 9:**
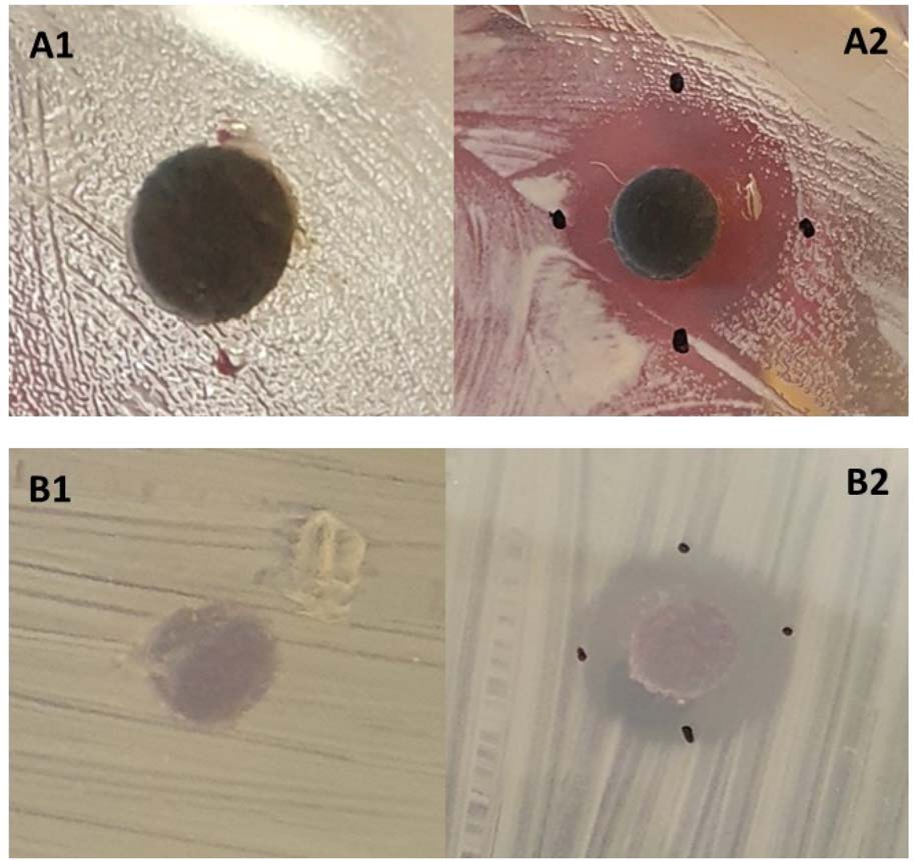
Absence (A1 and B1) and presence (A2 and B2) of growth inhibition zones surrounding S19-coated discs in *E. coli* (A) and *S. aureus* (B).

Bacteria incubated at 4 °C did not grow during the assay period. However, the coated-disc samples displayed growth inhibition halos against the selected bacteria at room temperature (Table 4 and Fig. 9). The package material with the bimetal-ion zeolite exhibited antimicrobial activity against the two bacteria. These preliminary positive results indicate that these bimetal-ion zeolite materials are very promising for application on an industrial level and could be a good solution to prolong the shelf life of fruits, ultimately reducing waste.

## Conclusions

Some pathogens from the global priority list were used to evaluate the antimicrobial activity of metal-ion zeolite materials based on LTA and MFI structures. Selected pathogens from the WHO global priority list were employed to evaluate the antimicrobial activity of metal-ion zeolite materials based on LTA and MFI frameworks. Our findings revealed clear structure–activity relationships, resulting in a distinct separation between the two zeolite types that demonstrated resilience against the pathogens studied. Particularly, the MFI structure exhibits the best results for bacteria and yeast strains, with lower amounts of ion-exchanged metals, regardless of whether mono- or bimetal-ion materials were employed. The LTA structure allows the best performance for bacterial population control. Regarding the clinical isolate strains, it was shown that the bimetal-ion zeolite materials are very effective at inhibiting the pathogens' growth.

Strain type and material concentration exhibited the strongest discriminative impact on antimicrobial efficacy, with clinical isolates showing higher susceptibility, and with lower material concentrations generally displaying higher activity. The growth phase and bacterial cell wall structure (Gram type) introduced observable but less pronounced variability, with exponential-phase and Gram-negative strains tending toward greater susceptibility under the tested conditions. Finally, this study demonstrates that multivariate statistics can effectively evaluate microbial growth inhibition without requiring a large set of microorganisms as susceptible indicator strains. This approach simplifies the assessment of the antimicrobial properties of the tested materials.

## Conflicts of interest

There are no conflicts to declare.

## Supplementary Material

RA-015-D5RA05465F-s001

## Data Availability

The data will be available upon request. Supplementary information: Tables S1 and S2 describe the metal-ion zeolite materials based in LTA and MFI structures prepared by the ion-exchange method; and bacterial and yeast strains used in this study, respectively. Tables S3–S5, display the dataset used for machine learning analysis. Fig. S1 describe the cluster map of the MIC values obtained for all LTA-based and MFI-based samples against the studied yeast species (2.5 indicates MIC > 2.0 mg mL^−1^). See DOI: https://doi.org/10.1039/d5ra05465f.
