# Supplementary material for: Antimicrobial agents based on metal-ion zeolite materials: a multivariate approach to microbial growth inhibition
Source: RSC Adv. 2025 Oct 1;15(43):36380–92. doi: 10.1039/d5ra05465f (PMC12486283; doi:10.1039/d5ra05465f)
Supplement: RA-015-D5RA05465F-s001 [file RA-015-D5RA05465F-s001.pdf]

Supplementary Information (SI) for RSC Advances.
This journal is © The Royal Society of Chemistry 2025

Supplementary Information

Antimicrobial agents based on metal-ion zeolite materials: a multivariate approach to microbial growth inhibition

Joana Guedes[a,b,&], Diogo B. Gonçalves[a,&], Catarina F. Rodrigues[a,b], Pier Parpot[a,c], António M. Fonseca[a,c], Cristina Almeida-Aguiar[b,*] and Isabel C. Neves[a,c,*]

---

[a]*Chemistry Centre of the University of Minho (CQ-UM), Department of Chemistry, University of Minho, Campus de Gualtar, 4710-057 Braga, Portugal.*

[b]*CBMA - Centre of Molecular and Environmental Biology, Department of Biology, University of Minho, 4710-057 Braga, Portugal.*

[c]*CEB - Centre of Biological Engineering, University of Minho, 4710-057 Braga, Portugal.*

[*]*corresponding authors: cristina.aguiar@bio.uminho.pt and ineves@quimica.uminho.pt*

[&]*authors with equal contribuition*

Table S1. Metal-ion zeolite materials based in LTA (Linde Type A) and MFI (Zeolite Socony Mobil-5) structures prepared by the ion-exchange method.

Samples	Label[a]	observations[b]	M (wt%)[c]
(NH_4)ZSM-5	S1	pristine zeolite used for the samples based in MFI	-
NaA	S2	pristine zeolite used for the samples based in LTA	-
**Modifications in LTA structure**			
Cu0.5A	S3	Cu^{2+} (0.5 mmol)	2.20
Ag0.5A	S4	Ag^{+} (0.5 mmol)	3.30
Ag2.5A	S5	Ag^{+} (2.5 mmol)	5.20
Ag2.5Cu0.5A	S6	S5 and Cu^{2+} (0.5 mmol)	5.10 (Ag)<br>4.50 (Cu)
Ag2.5Zn0.5A	S7	S5 and Zn^{2+} (0.5 mmol)	5.07 (Ag)<br>3.30 (Zn)
Ag0.5Zn2.5A	S8	S4 and Zn^{2+} (2.5 mmol)	2.10 (Ag)<br>4.00 (Zn)
Ag0.5Cu2.5A	S9	S4 and Cu^{2+} (2.5 mmol)	2.10 (Ag)<br>4.40 (Cu)
Ag0.5Cu0.5A	S10	S4 and Cu^{2+} (0.5 mmol)	0.70 (Ag)<br>2.00 (Cu)
Ag0.5Zn0.5A	S11	S4 and Zn^{2+} (0.5 mmol)	2.10 (Ag)<br>3.00 (Zn)
Cu0.5Ag0.5A	S12	S3 and Ag+ (0.5 mmol)	1.40 (Cu)<br>4.50 (Ag)
**Modifications in MFI structure**			
Ag0.5Zn0.5ZSM-5	S13	S19 and Zn^{2+} (0.5 mmol)	0.50 (Ag)<br>0.10 (Zn)
Ag0.5Cu0.5ZSM-5	S14	S19 and Cu^{2+} (0.5 mmol)	0.70 (Ag)<br>1.40 (Cu)
Ag0.5Zn2.5ZSM-5	S15	S19 and Zn^{2+} (2.5 mmol)	0.50 (Ag)<br>0.20 (Zn)
Zn0.5ZSM-5	S16	Zn^{2+} (0.5 mmol)	0.63
Cu0.5Ag0.5ZSM-5	S17	S18 and Ag^{+} (0.5 mmol)	0.60 (Cu)<br>0.73 (Ag)
Cu0.5ZSM-5	S18	Cu^{2+} (0.5 mmol)	1.20
Ag0.5ZSM-5	S19	Ag^{+} (0.5 mmol)	0.84

[a]Snumbers are the labels used to simplify the identification of the 19 zeolite samples tested throughout this work: 10 metal-ion zeolite materials prepared with the pristine zeolite LTA and 7 prepared with MFI;

[b]Metal nitrate solutions;

[c]Chemical analyses performed by ICP-AES.

Table S2. Bacterial and yeast strains used in this study (HSJP - Hospital de São João, Oporto; DBUM- Department of Biology, University of Minho).

	Bacteria	Strain	Origin	Label
Gram-negative	*Klebsiella pneumoniae (K. pneumoniae,* Kpl*)*		HSJP - *clinical isolate*	P2, *Kpl_{ci},*
	Proteus mirabilis (Pr. mirabilis, Pm*)*		HSJP - *clinical isolate*	P4, *Pm_{ci}*
	Pseudomonas aeruginosa (P. aeruginosa, Pa*)*		HSJP - *clinical isolate*	P5, *Pa_{ci}*
	Escherichia coli (E. coli, Ec*)*		HSJP - *clinical isolate*	P7, *Ec_{ci}*
		CECT423	DBUM - *type strain*	P8, *Ec_t*
Gram-positive	*Enterococcus cloacae (En. cloacae,* Enc*)*		HSJP - *clinical isolate*	P6, *Enc_{ci}*
	Enterococcus faecalis (En. faecalis, Enf*)*		HSJP - *clinical isolate*	P1, *Enf_{ci}*
	Staphylococcus saprophyticus (S. saprophyticus, Sts*)*		HSJP - *clinical isolate*	P3, *Sts_{ci}*
	*Methicillin-sensitive Staphylococcus aureus (*MSSA*)*		HSJP - *clinical isolate*	P9, *MSSA_{ci}*
		ATCC6538	DBUM - *type strain*	P10, *MSSA_t*
	*Methicillin-resistant Staphylococcus aureus (*MRSA*)*	DB1	DBUM - *clinical isolate resistant to methicillin*	P0, *MRSA_{ci}*
	**Yeast species**			
	Candida albicans (Ca)	1B	DBUM - *clinical isolate*	P11, *Ca_{ci}*
	Candida parapsilosis (Cp)	8A	DBUM - *clinical isolate*	P12, *Cp_{ci}*
	Candida glabrata (Cg)	8D	DBUM - *clinical isolate*	P13, *Cg_{ci}*
	Candida tropicalis (Ct)	28D	DBUM - *clinical isolate*	P14, *Ct_{ci}*
	Saccharomyces cerevisiae (S. cerevisiae, Sc*)*	BY4741 (*S288C-derivative*)	DBUM - *laboratory strain*	P15, *Sc_t*

Table S3. Minimum inhibitory concentration (MIC) values (mg/mL) for all zeolite samples against the tested microorganisms. Antimicrobial assays were performed with metal ion–zeolite samples at concentrations up to 2.0 mg/mL. MIC was reported as >2.0 mg/mL when microbial growth was observed at this maximum tested concentration. For data analysis, these cases were assigned a value of MIC = 2.5 mg/mL. Results are expressed exclusively as mean MIC value (standard deviation = 0 in all cases).

				MIC (mg/mL)															
	Metal ions (wt%)			P0	P1	P2	P3	P4	P5	P6	P7	P8	P9	P10	P11	P12	P13	P14	P15
Samples	Ag	Zn	Cu	MRSAci	Enfic	Kpl	Sts	Pm	Pa	Enc	Ec_{ci}	Ec_{t}	MSSAci	$MSSA_{t}$	Ca	Cp	Cg	Ct	Sc
S1	0	0	0	2.5	2.5	2.5	2.5	2.5	2.5	2.5	2.5	2.5	2.5	2.5	2.5	2.5	2.5	2.5	2.5
S2	0	0	0	2.5	2.5	2.5	2.5	2.5	2.5	2.5	2.5	2.5	2.5	2.5	2.5	2.5	2.5	2.5	2.5
S3	0	0	2.2	2.5	2.5	2.5	2.5	2.5	2.5	2.5	2.5	2.5	2.5	2.5	2.5	2.5	2.5	2.5	2.5
S4	3.3	0	0	2	2	2	2	2	2	2	2	2	2	2	2.5	2.5	2.5	2.5	2.5
S5	5.2	0	0	2	0.5	0.5	2	2	2	2	0.5	0.5	0.5	0.5	2.5	1	2.5	2.5	2.5
S6	6.8	0	4.5	0.5	0.5	0.5	0.5	0.5	0.5	0.5	0.5	0.5	0.5	0.5	2.5	2.5	2.5	2.5	2.5
S7	6.8	3.3	0	0.5	0.5	0.5	0.5	0.5	0.5	0.5	0.5	0.5	0.5	0.5	2.5	2.5	2.5	2.5	2.5
S8	2.1	4	0	0.5	0.5	0.5	0.5	0.5	0.5	0.5	0.5	0.5	0.5	0.5	2.5	2.5	2.5	2.5	2.5
S9	2.1	0	4.4	1	1	1	1	1	1	1	1	1	1	1	2.5	2.5	2.5	2.5	2.5
S10	0.7	0	2	1	1	1	1	1	1	1	1	1	1	1	2.5	2.5	2.5	2.5	2.5
S11	2.1	3	0	2	2	2	2	2	2	2	2	1	2	2	2.5	2.5	2.5	2.5	2.5
S12	4.5	0	1.4	0.5	0.5	0.5	0.5	0.5	0.5	0.5	0.5	0.5	0.5	0.5	2.5	2.5	2.5	2.5	2.5
S13	0.5	0.1	0	2.5	2.5	2.5	2.5	2.5	2.5	2.5	2.5	2.5	2.5	2.5	2.5	2.5	2.5	2.5	2.5
S14	0.7	0	1.4	2.5	2.5	2.5	2.5	2.5	2.5	2.5	2.5	2.5	2.5	2.5	2.5	2.5	2.5	2.5	2.5
S15	0.4	0.1	0	2.5	2.5	2.5	2.5	2.5	2.5	2.5	2.5	2.5	2.5	2.5	2.5	2.5	2.5	2.5	2.5
S16	0	0.63	0	2.5	2.5	2.5	2.5	2.5	2.5	2.5	2.5	2.5	2.5	2.5	2.5	2.5	2.5	2.5	2.5
S17	3.3	0	0.6	2	2	2	2	2	2	2	2	2	2	2	2.5	2.5	2.5	2.5	2.5
S18	0	0	1.2	2.5	2.5	2.5	2.5	2.5	2.5	2.5	2.5	2.5	2.5	2.5	2.5	2.5	2.5	2.5	2.5
S19	0.84	0	0	2.5	2.5	2.5	2.5	2.5	2.5	2.5	2.5	2.5	2.5	2.5	2.5	2.5	2.5	2.5	2.5

Strains in the green shaded columns (P7, P8 and P12) were selected for analysis in Figures 2A and 2B.

Table S4. Antimicrobial efficacy (%) of metal-ion zeolite samples prepared from both zeolite framework types (LTA: Linde Type A; MFI: Zeolite Socony Mobil-5) against bacterial strains

samples	Ag	Zn	Cu	P28	P29	P16	P17	P20	P21	P22	P23	P26	P27	P42	P43	P44	P45	P46	P47	P48	P49	P50	P51	P52	P53
S1	0	0	0	0	0	0	0	0	0	0	0	0	0	0	0	0	0	0	0	0	0	0	0	0	0
S2	0	0	0	0	0	0	0	0	0	0	0	0	0	0	0	0	0	0	0	0	0	0	0	0	0
S3	0	0	2.2	70	85	99	100	99	99	94	96	69	59	90	92	0	0	0	0	28	25	2	22	97	99
S4	3.3	0	0	100	100	98	100	97	100	97	100	86	100	42	100	0	100	68	61	0	100	0	0	0	100
S5	5.2	0	0	100	100	100	100	100	100	100	100	100	100	100	100	0	100	100	100	100	100	72	100	100	100
S6	6.8	0	4.5	100	100	100	100	100	100	100	100	100	100	100	100	100	100	100	100	100	100	100	100	100	100
S7	6.8	3.3	0	100	100	100	100	100	100	100	100	100	100	100	100	100	100	100	100	100	100	100	100	100	100
S8	2.1	4	0	100	100	100	100	0	100	100	100	100	100	100	100	100	100	100	100	100	100	100	100	100	100
S9	2.1	0	4.4	100	100	0	100	0	100	0	100	0	100	0	100	82	100	100	100	100	100	100	100	0	100
S10	0.7	0	2	100	100	100	100	100	100	0	73	87	100	100	100	0	100	100	100	100	100	0	100	100	100
S11	2.1	3	0	100	100	96	99	94	100	89	95	77	91	98	100	0	0	100	100	100	100	0	0	86	100
S12	4.5	0	1.4	100	100	100	100	100	100	100	100	100	100	100	100	100	100	100	100	100	100	100	100	100	100
S13	0.5	0.1	0	86	72	48	66	34	34	81	86	39	43	0	0	100	61	62	67	0	0	0	0	85	93
S14	0.7	0	1.4	45	17	74	23	74	71	81	53	54	47	0	0	10	15	43	73	28	20	68	64	99	98
S15	0.4	0.1	0	100	31	57	74	34	34	30	44	54	66	0	0	0	13	73	83	0	41	14	100	88	87
S16	0	0.63	0	72	86	92	74	90	96	53	44	77	77	0	0	0	23	73	83	45	46	89	87	92	85
S17	3.3	0	0.6	0	100	57	70	0	60	0	0	50	80	0	30	42	100	0	62	0	95	0	100	0	70
S18	0	0	1.2	0	0	0	0	0	0	0	0	0	0	0	0	0	13	0	0	0	0	0	0	0	0
S19	0.84	0	0	86	86	57	100	95	95	11	30	47	51	0	41	17	20	54	79	38	49	41	25	98	98

Table S5. Antimicrobial efficacy (%) results of a posteriori balanced metallic zeolite-based sample set, used to assess the contribution of strain type. This dataset is a transposed subset of the original one.

Material conc.	Type strain	Growth phase	Gram type	LABELS	S1	S2	S3	S4	S5	S6	S7	S8	S9	S10	S11	S12	S13	S14	S15	S16	S17	S18	S19
-1	-1	1	-1	0.5 mg/mL EC_t	0	0	99	97	100	100	100	0	0	100	94	100	34	74	34	90	0	0	95
1	-1	1	-1	1 mg/mL EC_t	0	0	99	100	100	100	100	100	100	100	100	100	34	71	34	96	60	0	95
-1	-1	1	1	0.5 mg/mLMSSA$_t$	0	0	69	86	100	100	100	100	0	87	77	100	39	54	54	77	50	0	47
1	-1	1	1	1 mg/mL $MSSA_t$	0	0	59	100	100	100	100	100	100	100	91	100	43	47	66	77	80	0	51
-1	1	-1	-1	0.5 mg/mL ECci	0	0	100	94	100	100	100	100	86	0	0	100	83	67	87	92	0	0	82
1	1	-1	-1	1 mg/mL ECci	0	0	73	100	100	100	100	100	100	73	0	100	79	42	87	88	75	0	81
-1	1	-1	1	0.5 mg/mLMSSA$_{Ci}$	0	0	100	94	100	100	100	100	86	0	0	100	83	67	87	90	0	0	82
1	1	-1	1	1 mg/mL $MSSA_{ci}$	0	0	73	100	100	100	100	100	100	73	0	100	80	45	88	85	65	0	81

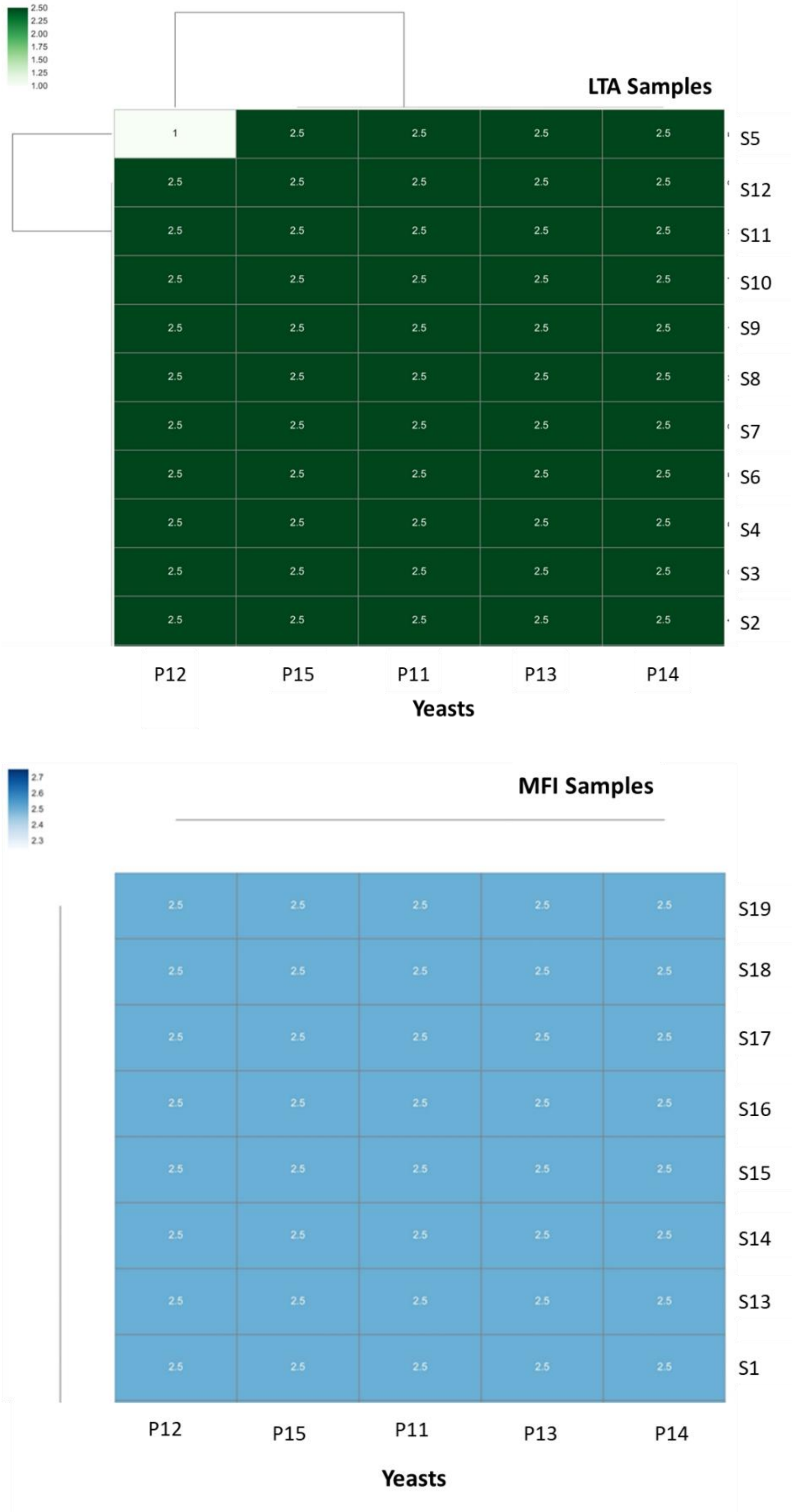

Figure S1. Cluster map of the MIC values obtained for all LTA-based (Linde Type A) and MFI-based (Zeolite Socony Mobil-5) samples against the studied yeast species (2.5 indicates MIC > 2.0 mg/mL).
